# Fast, Multiple-Use Optical Biosensor for Point-of-Care Glucose Detection with Mobile Devices Based on Bienzyme Cascade Supported on Polyamide 6 Microparticles

**DOI:** 10.3390/polym15132802

**Published:** 2023-06-24

**Authors:** Joana F. Braz, Nadya V. Dencheva, Shafagh D. Tohidi, Zlatan Z. Denchev

**Affiliations:** 1 IPC-Institute for Polymers and Composites, University of Minho, 4800-056 Guimarães, Portugal; joanabraz@dep.uminho.pt; 2 DTx CoLab-Digital Transformation CoLab, University of Minho, 4800-056 Guimarães, Portugal; shafagh.tohidi@dtx-colab.pt

**Keywords:** enzyme immobilization, GOx/HRP enzyme cascade, glucose detection, polyamide microparticles, sensors and actuators

## Abstract

Non-invasive glucose determination provides major advantages in health monitoring and protection. It enables widespread point-of-care testing, which is affordable, sensitive, specific, rapid and equipment-free. This work reports on the development and analytical performance of a colorimetric biosensor in detecting glucose in human urine. Highly porous polyamide microparticles were synthesized as the support for the glucose oxidase (GOx) and horseradish peroxidase (HRP) dyad, which was immobilized randomly or consecutively—first HRP and then GOx. The latter system was superior, as GH@PA-C showed much higher activity than the random system, and it was used to prepare the biosensor, along with the 3,3′,5,5′-tetramethylbenzidine chromogen. When in contact with urine, the biosensor displayed a strict linear correlation between the color difference and the glucose concentration in urine in the range of 0.01–3.0 mM, as established by the CIELab image processing algorithm and UV-VIS measurements. The biosensor acted in 20 s and had a detection limit of 30.7 µM in urine, high operational activity at pH = 4–8 and unchanged detection performance after 30 days of storage. Its unique feature is the possibility of multiple consecutive uses without the serious deterioration of the recovery and dispersion values. These characteristics can open the way for new routines in non-invasive personal diabetes detection.

## 1. Introduction

The control of blood glucose levels is one of the essential homeostatic mechanisms in the human body and is achieved by hormonal, nutrient-related or neuronal cues. Any deregulation in this fine-tuned system causes severe medical complications, including diabetes mellitus, renal failure, heart disease or even cancer [1]. Tracking glucose levels in the blood or other human body fluids is currently the most effective disease marker. 

There exist several conventional tests to measure blood glucose [2] requiring invasive blood collection, which causes discomfort to patients. The results have to be processed in specialized labs [3], making the continuous monitoring of blood glucose difficult [4]. An alternative non-invasive glucose determination method presents major advantages: e.g., it is affordable, sensitive, specific, rapid and equipment-free [5]. This makes widespread, secure and accurate point-of-care testing (POCT) possible. 

Analyzing human body fluids is considered an important step toward non-invasive glucose POCT. Interstitial fluid, urine, sweat, ocular fluids and saliva also contain glucose, which, due to significant advances in technology, has become accessible for accurate, stable and fast determination [6]. There exist two general analytical methods for studying glucose levels that can be applied in POCT: optical (colorimetric) [7,8] and electrochemical [9,10]. Although electrochemical detection is more sensitive, colorimetric methods are more acceptable because their procedures are simple and the result can be observed intuitively, even with the naked eye [6,11].

The steady demand for cheaper, more sensitive and easy-to-use glucose sensors for POCT has led to the development of numerous types of biosensors. Most of them are based on the immobilization of the bioreceptor on or in an appropriate support, inorganic or organic in nature, with the latter also including polymers [12,13,14]. 

The majority of optical biosensors developed so far have employed the known cascade reaction of glucose oxidation by the glucose oxidase/horseradish peroxidase (GOx/HRP) enzyme dyad in the presence of the chromophore transducer 3,3′,5,5′-tetramethlbenzidine (TMB). Thus, Sun et al. used organic–inorganic nanoflowers (NFs) containing GOx/HRP to determine the glucose content [15] and added TMB separately to the analyzed urine sample. A linear response was found in the range of 0.5–50 µM glucose, with the limit of detection (LOD) being 0.2 µM. More recently, the same GOx/HRP NFs were employed in a paper-based device for glucose detection and quantification [16]. TMB in combination with chitosan was used for colorimetric glucose quantification using the HSV color space analysis of digital photographs. The detector response was linear up to a 300 µM glucose concentration, with a LOD value of 15.6 µM and a time to complete the assay of 15 min. In the work of Luo et al. [17], the GOx/HRP dyad was chemically bound to a cellulose membrane, and the resulting biosensor was used to test glucose in urine. Again, the TMB transducer was separately added to the test sample. The color changes were collected with a digital camera and analyzed using image processing software. The LOD of this biosensor was 0.45 mM in the linear range of glucose concentrations between 1 and 11 mM, with the time of the assay being 5 min. 

An innovative biosensor for glucose based on the GOx/HRP cascade was prepared by the immobilization of the two enzymes on a poly (aniline-*co*-anthranilic acid) copolymeric film that also serves as a colorimetric transducer [18]. A smartphone employing the free ColorLab^®^ Android application was used for glucose concentration quantification. A linear response for this biosensor was observed for glucose concentrations of up to 200 µM, with a LOD of 14–16 µM. Another copolymeric support based on poly(vinyl acetate)-co-polyethylene (PVA/PE) porous strips was also tested in glucose biosensors [19]. Thus, GOx/HRP/Mn_3_(PO_4_)_2_ organic–inorganic NF structures were directly “planted” on the PVA/PE copolymer, along with the TMB chromogen. The testing of glucose solutions revealed a linear response between 0.25 and 10 mM with a LOD of 0.14 mM. The color response was detected from digital photographs by means of the CIELab system. 

An interesting approach using the GOx/HRP system for glucose biosensors was the immobilization of the enzyme dyad directly upon the particles of the TMB chromogen [20]. This biosensor with non-covalent enzyme immobilization displayed good performance for colorimetric and photothermic assays of glucose in a range of concentrations up to 100 mM, with LOD values of 12 and 28 µM, respectively. Tang et al. [21] recently demonstrated that some mesoporous hydrogen-bonded organic frameworks can also be good supports for the GOx/HRP dyad. Using a TMB colorimetric read-out, these biosensors showed linearity between 10 and 2000 µM. 

Summarizing the above, the GOx/HRP enzyme cascade with the TMB chromogen is the most frequently used system for optical glucose biosensors. Their color change can be documented in smartphone digital photographs and then quantified by standard image processing software. This expands mobile phone technology to POCT glucose detection [22].

Based on the existing research on glucose biosensors, it can be hypothesized that other properly structured and shaped polymer supports could also be used for the immobilization of the GOx/HRP bienzyme cascade. Thus, the activated anionic ring-opening polymerization (AAROP) of lactams was previously applied by us to produce porous polyamide microparticles [23,24]. Chemically, polyamides and all protein-based biomolecules are structural analogs, which permits multiple hydrogen bonds to form between them, thereby making effective non-covalent immobilization possible. Thus, laccase-immobilized polyamide 6 (PA6) microparticles were used for the development of an optical biosensor for catechol in effluents with a LOD of 11 µM, responding linearly up to 118 µM catechol [25]. 

This work is the first report on the application of PA6 microparticles as carriers for the GOx/HRP cascade and the use of this system as a glucose biosensor in urine samples. Microparticles with a controlled shape, size and porosity were synthesized by AAROP in suspension. GOx and HRP were non-covalently immobilized on the particles via adsorption from aqueous solutions, applying different orders and concentrations of the reagents. To build the biosensor, PA6 microparticles containing the proper concentrations of the GOx/HRP dyad and TMB were fixed on inert strips by means of a water-permeable segmented PA6-polyether block copolymer. After studying the activity of the sensor and calibrating it with known amounts of glucose, real human urine samples were studied. In all cases, the color changes of the biosensor were quantified by both UV/VIS spectrometry and the digital processing of color images. The results obtained confirmed the high potential of PA6 microparticles as suitable carriers of the GOx/HRP cascade for POCT in glucose biosensors.

## 2. Materials and Methods

### 2.1. Materials and Reagents

The ɛ-caprolactam (ECL) monomer with reduced moisture content for anionic polymerization (AP-Nylon^®^) was purchased from Brüggemann Chemical (Heilbronn, Germany). Before use, it was kept under vacuum for 1 h at 23 °C. As a polymerization activator, Brüggolen C20^®^ (C20) from the same company was used. The initiator sodium dicaprolactamato-bis-(2-methoxy-ethoxo)-aluminate (Dilactamate^®^, DL, 85% solution in toluene) was purchased from Katchem (Prague, Czech Republic) and applied without further treatment. Toluene, xylene, methanol and other solvents were all of analytic grade, purchased from Merck (Lisbon, Portugal) and used as received. GOx from *Aspergillus Niger* type VIII, D–(+) glucose (*purum p.a*.), NaH_2_PO_4_·2H_2_O, 99% (*purum p.a.*), Na_2_HPO_4_·2H_2_O, (98%), NaCl (99%) and CH_3_COOH (99%) were purchased from Merck/Sigma Aldrich, Lisbon, Portugal. HRP from *Amoracia rusticana* and CH_3_COONa (99%) were purchased from Alfa Aesar (Lancashire, UK). 3,3′,5,5′-Tetramethylbenzidine (TMB, 99%) was purchased from Acros Organic (Porto Salvo, Portugal). Pebax MH 1657 resin (block copolymer with 60% soft polyethylene oxide segments and 40% PA6 hard segments) was kindly donated by Arkema (Serquigny, France), and poly(ethylene terephthalate) film (Melinex^®^) was supplied by Addev Materials (Lyon, France).

### 2.2. Characterization Methods

Scanning electron microscopy (SEM) studies were performed using a NanoSEM-200 apparatus from FEI Nova (Hillsboro, OR, USA) using mixed secondary electron/back-scattered electron in-lens detection. All the samples were observed after sputter coating with Au/Pd alloy in a 208 HR instrument from Cressington Scientific Instruments (Watford, UK) with high-resolution thickness control. 

UV-VIS absorbance spectral measurements were carried out using a 2401PC double-beam spectrophotometer from Shimadzu (Tokyo, Japan) with a thin-film holder that enables the interrogation of the sensing area.

Synchrotron wide- (WAXS) and small-angle X-ray scattering (SAXS) measurements were performed at the NCD-SWEET beamline of the ALBA synchrotron facility in Barcelona, Spain. Two-dimensional detectors were used, namely, LH255-HS (Rayonix, Evanston, IL, USA) and Pilatus 1 M (Dectris, Baden Daettwil, Switzerland), to register the WAXS and SAXS patterns, respectively. The sample-to-detector distance was set to 111.7 mm for WAXS and 2700 mm for SAXS measurements, with the λ of the incident beam being 0.1 nm and the beam size being 0.35 mm × 0.38 mm (h × v). The 2D data were reduced to 1D data using pyFAI software version 0.21.3 [26]. For the processing of WAXS and SAXS patterns, the commercial package Peakfit 4.12 (2016) by SYSTAT (San Jose, CA, USA) was implemented.

### 2.3. Synthesis of PA6 Microparticles

The PA6 microparticles (PA6 MPs) were synthesized by activated anionic ring-opening polymerization (AAROP), as previously described by Dencheva et al. [23,25]. First, 0.5 mol of ECL was added to 100 mL of a mixed hydrocarbon solvent (toluene/xylene 1:1 by volume) while stirring under a nitrogen atmosphere, and the reaction mixture was refluxed for 10–15 min. Subsequently, 3.0 mol% of DL and 1.5 mol% of C20 were introduced simultaneously. The reaction time was 1 h from the point of the catalytic system addition, and the temperature was maintained in the 125–135 °C range with constant stirring at about 800 rpm. The final PA6 MPs were formed as a fine powder that was separated from the reaction mixture by hot vacuum filtration, washed several times with methanol and dried for 30 min in a vacuum oven at 60 °C. To remove the low-molecular-weight PA6 fractions, further Soxhlet extraction for 4 h with methanol was employed. The resulting neat PA6 MPs were kept in a desiccator for further treatment. The scheme of PA6 MP synthesis by AAROP is presented in Figure S1 of the Supplementary Materials. 

### 2.4. Immobilization of GOx and HRP on PA6 MPs

A typical immobilization process was carried out by first preparing the enzyme solutions in double-distilled water: GOx solution with a concentration of 1 mg/mL and HRP solution with a concentration of 0.5 mg/mL. These optimal enzyme concentrations were established in a previous work [14]. Thus, two types of non-covalent immobilization were performed via the adsorption of the enzymes on PA6 MPs: (i) random co-immobilization and (ii) consecutive immobilization. In random co-immobilization, 50 mg of PA6 MPs was introduced into a mixture of 2.5 mL of the GOx solution and 2.5 mL of HRP and incubated at 37 °C for 24 h while agitating in a laboratory orbital shaker. The resulting random polyamide/bienzyme complex was designated as GH@PA-R. For the preparation of the conjugates with consecutive immobilization, 50 mg of PA6 MPs was first introduced into 5 mL of the HRP solution and incubated at 37 °C for 6 h. The supernatant was then decanted and stored for subsequent use. Thereafter, a 5 mL aliquot of the GOx solution was added to the PA6/HRP conjugate and incubated for an additional 18 h at 37 °C. The bienzyme complex made in this way was designated as GH@PA-C. 

After incubation was complete, both sample types were centrifuged, the supernatant was decanted and stored for further analysis, and the final PA-GOx/HRP systems were washed two times with double-distilled water to remove non-immobilized enzymes. 

### 2.5. Determination of the Total Amount of Protein in PA6 Microparticles

After incubation, the supernatants were subjected to UV analysis to determine the GOx and HRP contents immobilized and calculate the amount of the total protein (TP) incorporated into the PA6 MPs, expressed as:

(1) $$TP=C_{0}-C_{S}$$

where C_0_ is the starting protein content, and C_S_ is the protein content in the resultant supernatant after immobilization. To estimate C_S_, the direct UV quantification of the absorption peak at λ ≈ 278 nm was performed. This peak (often referred to as A280) is ascribed to the benzene rings of phenylalanine, tryptophan and tyrosine residue units of proteins. This direct method produces reliable data with a standard deviation of 3–5%, while the indirect Bradford and bicinchoninic acid colorimetric assays for protein content result in data dispersions of up to 15%. Hence, the absorbance at λ ≈ 278 nm was measured, and C_S_ was calculated using a standard calibration plot [14].

### 2.6. Activity Measurements by UV-VIS

To investigate the activity of the free GOx/HRP bienzyme system on a certain amount of glucose, 0.1 mL of a 0.1 wt.% TMB ethanol solution, 0.015 mL of GOx (1.0 mg/mL), 0.005 mL of HRP (0.5 mg/mL) and 0.016 mL of 5 mM glucose were added to 1.864 mL of acetate buffer (0.2 M, pH 4). The time-dependent UV-VIS absorbance of this mixture at 652 nm was studied within a period of 2 min. In the case of the MP-supported enzyme dyads GH@PA-R and GH@PA-C, a similar procedure was used, in which, instead of the free enzymes, 5 mg of each of the wet immobilized complexes was used. In the absorbance vs. time plot, the linear relationship within the 0–15 s region was considered to determine the initial rate. The absolute activity was expressed in µkatals, whereby 1.0 µkat is the amount of the enzyme required to convert 1.0 µmol of TMB to the oxidized colored product in 1 s at pH 4 and a temperature of 20–23 °C. To enable comparison, two more activities were also calculated: the specific activity, which is the absolute activity per 1 mg of protein, and the relative activity, which expresses the activity of the immobilized systems relative to that of the free enzyme dyad, with the latter being 100%. 

The activities of both free and immobilized enzymes were also studied as indicated above in the pH interval from 4 to 8, always using 0.2 M buffers, as follows: acetate buffer for pH 4, citrate buffer for pH 5 and 6, and phosphate buffer for pH 7 and 8. 

### 2.7. Biosensor Construction and Use

**Figure 1:**
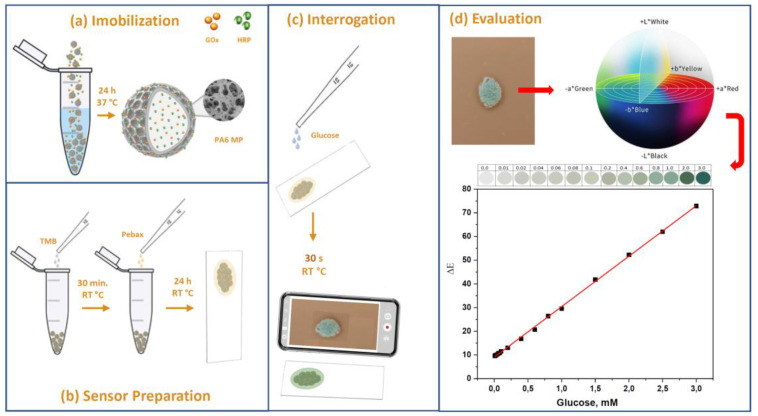
Schematic representation of the various phases of sensor preparation, sensor interrogation and results evaluation. For more details, see the text.

The entire process of biosensor preparation, interrogation and data processing is presented in Figure 1. 

Biosensors were produced on 70 µm thick transparent PET film supports cut into 40 mm × 20 mm strips in several steps. First, 5 wt.% Pebax^®^ in a methanol/water solution (70:30 vol/vol) was prepared by refluxing the copolymer granules at 80 °C and cooling them down to room temperature. The chemical structure of the segmented Pebax block copolymer is presented in Figure S2. Next, 50 mg of PA6 MPs with immobilized GOx and HRP, designated as GH@PA-C (Figure 1a), was mixed with 0.120 mL of 2 wt.% TMB solution and left to dry at room temperature in a light-protective container. Then, the particulate system so obtained was suspended in 0.250 mL of the Pebax solution. For the casting of the biosensors, approximately 2 mg of this suspension was deposited upon the PET strips in circular interrogation areas with approximately 3–4 mm diameter (Figure 1b). The final sensor strips were left to dry in a methanol/water atmosphere at 5 °C for 24 h in a light-protective container.

In the interrogation step (Figure 1c), the sensor was brought into contact with a water solution or urine with a certain glucose concentration [G]. The color change started immediately, and after 20 s, it was registered by digital photography and UV/VIS spectrometry as a reference. The digital photographs were analyzed using the CIELab coordinates to produce the $\Delta E=f\left[G\right]$ calibration graph (Figure 1d). For the UV/VIS measurement, the absorption at λ = 652 nm was plotted against the glucose concentration [G] to construct the respective calibration curve. 

### 2.8. Detection of Glucose from Smartphone Digital Images

Image acquisition was performed in a portable light box photography studio with an orange background and reproducible white light illumination. A Xiaomi T11 Pro smartphone with a 5 MP Samsung S5K5E9 1/5” sensor was used with a pixel size of 1.12 µm and a 50 mm f/2.4 lens. The biosensor strips were placed horizontally inside the box at the designated position. Photographs were taken from the top of the box through a designated window, with the distance between the sensor and the camera lens being 5 cm. First, a blank image (the sensor without glucose) was taken to serve as a reference. Then, 0.03 mL samples of glucose substrate solutions with varying concentrations were pipetted onto the interrogation area and left to actuate for 20 s, after which the final photograph of the colored sensor was taken. In this way, the range of 0.01–10 mM glucose was studied in aqueous solutions. 

The color change was determined in two ways: UV-VIS and by Photoshop. For the UV/VIS measurement, the GOx/HRP biosensors, after their color change, were placed vertically in the sample holder, and the absorbance at 652 nm was measured in the area of the interrogation zone. For the Photoshop evaluation, the digital images before and after the color change were processed according to the method described by Luo et al. [19], which employs the following equation:

(2) $$\Delta E=\sqrt{{{(L}_{1}^{*}-L_{0}^{*})}^{2}+{\left(a_{1}^{*}-a_{0}^{*}\right)}^{2}+{\left(b_{1}^{*}-b_{0}^{*}\right)}^{2}}$$

where L*, a* and b* are the values from the CIELab color system that characterize the color along three axes: L* is the lightness and ranges from black (0) to white (100), a* varies from green to red, and b* ranges from blue to yellow. The subscripts 0 and 1 represent the sensor area color before and after glucose addition, so ΔE quantifies the cumulative color change. Standard curves using both UV/VIS and Photoshop methods covering the 0.01–10 mM glucose range were constructed.

### 2.9. Detection of Glucose in Real Urine Samples

Biosensors based on the GH@PA-C bioreceptor were studied for detecting glucose in real human urine. Freshly collected urine samples were stored at –8 °C. Before testing, these samples were kept for 2 h at room temperature and centrifuged at 5000 rpm for 30 min. The urine supernatant was used to prepare glucose solutions with concentrations between 0.01 and 10 mM. Then, 0.03 mL of each of these solutions was pipetted onto the interrogation area of biosensors and placed horizontally in the designated area of the light box studio. After 20 s, the color change took place, and a digital photograph was taken to be analyzed later, as indicated in the previous section. A control test was carried out using distilled water as a blank sample. Right after that, the colored biosensor was placed in a UV-VIS spectrometer, and the absorbance at *λ_max_* = 652 nm was also measured for comparison. These measurements were used to construct a standard calibration curve covering the range of 0.01–10 mM glucose. 

### 2.10. Glucose Biosensor Shelf Life and Multiple Uses

The storage stability of the biosensors based on the GH@PA-C bioreceptor was studied in the following way. The biosensors were kept at room temperature inside an air-sealed and UV-light-protective container for 30 days. Every 7 days, biosensor strips were applied to urine with known glucose concentrations (0.04 mM, 0.4 mM and 2 mM) employing the protocol in Section 2.9. To test the possibility of multiple uses, 30 min after the first measurement, an additional measurement with the same biosensors was performed. This procedure was repeated up to 5 times. 

## 3. Results and Discussion

### 3.1. Morphological and Structural Characterization 

**Figure 2:**
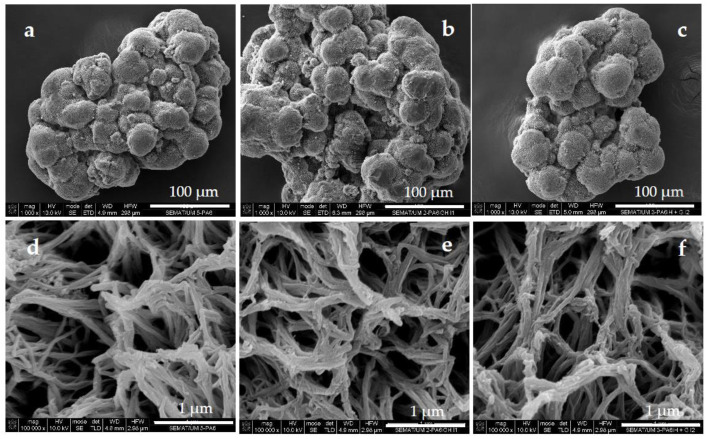
SEM micrographs of (**a**) PA6 MPs; (**b**) GH@PA-R sample (simultaneous GOx and HRP immobilization, random adsorption); (**c**) GH@PA-C sample (first immobilization of HRP, followed by GOx immobilization). Micrographs (**d**–**f**) represent closer views of samples (**a**–**c**).

The first step of the biosensor preparation was the synthesis of PA6 MPs via the AAROP of ε-caprolactam, as explained in detail in earlier studies [23,25], employing DL as the anionic initiator and C20 as the activator according to the reaction scheme in Figure S1. As seen in the SEM micrographs in Figure 2a,d, the resulting microparticles had maximum dimensions in the range of 100 µm and, under close observation, displayed a highly porous, scaffold-like morphology with submicron-size open pores. The adsorption immobilization of GOx and HRP (simultaneously in GH@PA-R or first HRP and then GOx in GH@PA-C complexes) did not produce any differences in the sample morphology that could be directly observed by SEM. 

**Figure 3:**
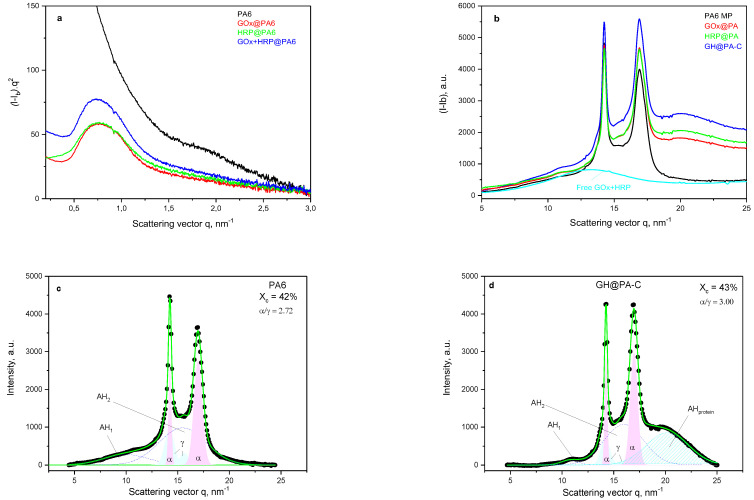
Synchrotron X-ray scattering studies of enzymes immobilized on PA MP supports: (**a**) Lorentz- and background-corrected linear SAXS profiles; (**b**) background-corrected linear WAXS profiles; (**c**) deconvolution of neat PA6 support WAXS profile; (**d**) deconvolution of the GH@PA-C WAXS profile. All patterns were obtained at room temperature. For sample designations, see Section 2.4.

The use of synchrotron SAXS (Figure 3a) allowed further clarification of the structure of the PA6-supported enzyme dyad. This method probes density periodicities with dimensions in the 2–25 nm range, which includes the sizes of the crystalline lamellae typically found in semicrystalline polymers. The appearance of SAXS peaks depends on the phase contrast between the crystalline and amorphous domains of the lamellar systems. Figure 3a displays the background- and Lorentz-corrected linear SAXS profiles of the empty PA6 MP support compared to the patterns of the GOx@PA, HRP@PA and bienzyme GH@PA-C samples. The last pattern completely coincides with that of the GH@PA-R sample. 

The absence of a periodicity peak in the pattern of the empty PA6 MP support could be explained by the porosity of the microparticles, which eliminated the phase contrast between the amorphous and crystalline domains. Whenever GOx, HRP or both of them were immobilized on MPs, SAXS peaks were registered with long spacings *L* = 8.0 nm (PA6 with individual GOx or HRP) or *L* = 8.6 nm (GH@PA-C). This means that the immobilized enzymes enter the nanosized pores of the MP support, thus significantly improving the phase contrast and causing the appearance of SAXS periodicity peaks. Similar effects were observed in the SAXS patterns of PA6 MPs containing adsorption-immobilized laccase [25]. Notably, the SAXS method cannot distinguish between the GH@PA-R and GH@PA-C samples. 

As seen in Figure 3b, the neat PA6 MPs present the well-known WAXS pattern of PA6, with its two strong reflections at *q* = 14.4 nm^−1^ and 17.2 nm^−1^ ascribed to the monoclinic unit cell of α-PA6 with d[200] = 4.36 Å and d_[002/202]_ = 3.65 Å. All other PA6 samples in Figure 3b with immobilized enzymes contain these two reflections with the same intensity ratio and angular positions. This is an indication that the immobilization of GOx, HRP or both of them on PA6 MPs did not affect the crystalline structure of the MP carrier. Notably, the mixed GOx + HRP solid enzymes produced a diffuse amorphous peak at *q* = 14.0 nm. In the samples containing one or both of the enzymes, this halo shifts to higher values of the scattering vector *q*, causing a significant increase in the background level. 

The deconvolution of the WAXS profiles of the empty PA6 support and the GH@PA-C complex (Figure 3c,d respectively) confirmed the finding that enzyme immobilization does not significantly change the crystalline structure of the PA6 support, i.e., its degree of crystallinity *X_c_* and polymorph content α/γ, with the small difference being within the limits of the experimental error. At the same time, a diffuse peak related to the immobilized enzymes appears at *q* = 20 nm (Figure 3d), so a shift of 6 nm as compared to the non-immobilized dyad (Figure 3b) is present. Since the amorphous halo in WAXS is related to intermolecular interactions [27], its position must be dependent on the degree of packing of the molecules (i.e., the density) of the respective amorphous phase (in this case, that of the GOx + HRP enzymes), where the larger the value of the scattering vector *q*, the smaller the intermolecular distance between enzyme macromolecules and, consequently, the higher their density. This means that the adsorbed dyad in the GH@PA-C sample and single enzymes in the GOx@PA and HRP@PA samples have a denser packing as compared to the free enzymes. This is presumably attributed to the intensive formation of multiple H-bonds between the enzymes and PA6 macromolecules in the amorphous phase of the support, which is due to the nature of the non-covalent immobilization. 

### 3.2. Description of the GOx-HRP Cascade Reaction

The biosensor in this work is based on the known cascade reaction of glucose oxidation by the GOx/HRP enzyme dyad in the presence of the TMB chromophore transducer, which is a typical substrate for HRP (Figure S3 of the Supplementary Materials).

In this cascade reaction, GOx catalyzes the oxidation of β-D-glucose to release D-glucono-δ-lactone and H_2_O_2_. As pointed out by Josephy et al. [28] and later confirmed by Misono et al. [29], H_2_O_2_ is used by HRP to oxidize the native diamine TMB, forming the cation-free radical compound TMB^●+^. Thereafter, the latter forms a blue-green-colored charge-transfer complex [TMB…DTMB] detectable by UV/VIS at *λ_max_* = 652 nm. Here, DTMB is the diimine derivative of TMB. The final oxidation product of the cascade reaction is the bi-cation TMB^2+^ with *λ_max_* = 450 nm and a yellow color. Since both H_2_O_2_ formation and TMB oxidation have similar reaction rates, the overall reaction rate of this cascade process will depend on how quickly and effectively H_2_O_2_ reaches HRP. Therefore, the overall reaction rate will depend on the distance between GOx and HRP and their special orientation, i.e., on the architecture of the co-immobilization of the bienzyme complex [30].

### 3.3. Comparative Activity Studies

**Figure 4:**
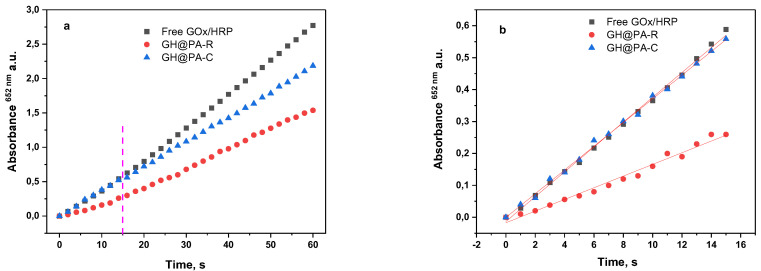
Overall catalytic activities (0.2 M acetate buffer, pH 4) of GH@PA-R and GH@PA-C complexes in comparison to the free GOx/HRP dyad in two different time intervals: (**a**) 0–60 s; (**b**) 10–15 s. For more details, see the text.

The overall catalytic activities of both enzymatic systems prepared with either the random immobilization of the GOx/HRP dyad (GH@PA-R) or the controlled localization of enzymes (GH@PA-C) were assessed and compared to that of the free GOx/HRP system. For this purpose, the enzymatic cascade reaction in Figure S3 was used. Figure 4 displays the time dependence of the absorbance at λ = 652 nm. The linear segment of the curves in the range of 0–15 s was considered to determine the initial rates and to calculate the catalytic activity of each enzymatic system. More data about the fitting paraments of the line graphs can be found in Figure S4 of the Supplementary Materials. Table 1 allows the comparison of the activity parameters of all bienzyme systems of this study determined under the same conditions, as mentioned in Section 2.6 of the Materials and Methods.

The total protein content in Table 1, determined according to Equation (1), was used to normalize the slope of the linear dependencies in Figure 4, which allows the comparison of the activity data. The specific activities for the three samples were calculated in µkat. L^−1^ or in activity units (U). The last column in Table 1 presents the relative activities, wherein that of the free GOx/HRP system is considered 100%.

Figure 4 and the data in Table 1 show that the catalytic activities based on the initial rates of the free GH dyad and of the GH@PA-C sample almost coincide, while GH@PA-R, with the random immobilization of GOx and HRP, displays only half of this activity. In the 20–60 s time interval, the reaction rate of the GH@PA-R sample starts lagging behind that of the free enzymes but still continues to be better than that of the GH@PA-R sample. 

The different activities of the GH@PA-C and GH@PA-R complexes show that the location and mutual organization of the enzymes on the PA MP support do really matter for the overall rate of the reaction. Apparently, the enzymes´ arrangement in GH@PA-C is much more advantageous for the cascade reaction to occur effectively. In GH@PA-R, immobilization takes place from a solution of the GOx/HRP mixture, leading to a random distribution of both enzyme molecules within the porous support. In the case of GH@PA-C, HRP is first immobilized on the MPs, the resulting HRP@PA complex is removed and then placed into the GOx solution to immobilize the second enzyme. Thus, in GH@PA-C, there is some stratification and alignment of the enzymes: HRP is located deeper in the MP pores, while GOx is closer to the MP surface. Most likely, the two enzymes are in separate domains within the pores but close to one another. Such an arrangement favors the cascade reaction of glucose detection because, as seen in the reaction scheme in Figure S3, GOx enters the reaction first. Thereafter, the low-molecular-weight H_2_O_2_ produced migrates deeper into the MPs to reach the HRP-containing domains. The final result is the oxidation of TMB, which is randomly distributed within the whole MP volume, and the production of the colored charge-transfer complex in an amount proportional to the glucose concentration. Thus, the GH@PA-C system was selected for further studies and sensor preparation.

### 3.4. Detection of Glucose in Water Solutions

**Figure 5:**
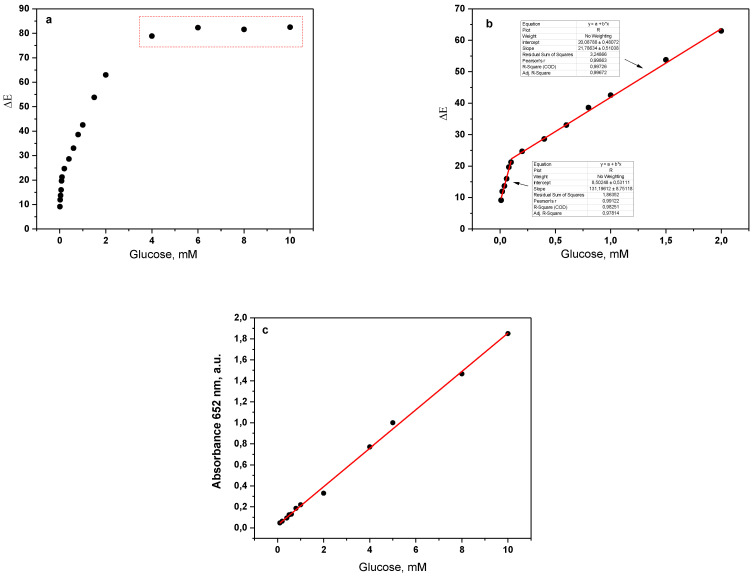
Relationship between color change and glucose concentration in water solution tested by GH@PA-C sensor by means of (**a**,**b**) processing of smartphone digital photographs; (**c**) UV/VIS reference measurement. Every data point is a mean of at least three trials.

Initially, the biosensor was tested with aqueous solutions of glucose, varying the concentrations in a broad range. The color change was monitored for 60 s. As expected, the interrogation area changed from colorless to blue-green, and the color parameters stabilized within the next 20 s. Thus, the digital images taken at the 20th second were used to calculate the color change ΔE according to Equation (2). Figure 5a shows the plot of ΔE vs. glucose concentration in the 0–10 mM range. 

Figure 5a shows that the color change of the sensor was linearly proportional to the glucose concentrations up to 2 mM, after which saturation occurred. Two distinct linear intervals were clearly observable (Figure 5b): from 0.01 to 0.1 mM with a larger slope (i.e., higher sensitivity) and from 0.1 to 2.0 mM with a lower slope value. In both cases, high coefficients of linear regression (R^2^ ≈ 0.99) were achieved. Right after being photographed, all treated sensors were placed in a UV/VIS spectrometer to obtain their absorption spectra in the 400–800 nm range as a function of glucose concentration (Figure 5c). In this case, the dependence was strictly linear in the whole 0.01–10 mM range. Evidently, spectroscopic methods display broader operational ranges than the processing of digital images. Our results also show that the processing of smartphone photographs can provide a good trade-off between the easier, equipment-free quantification of the color change and the sufficient range of linear response.

Thus, an upper limit of 2.0 mM glucose (36 mg/dL) is sufficient for practical applications because glucose levels of 0–25 mg/dL (0.00–1.35 mM) in urine are typical for a healthy person, and therefore, any higher value would be an indication to proceed with more sophisticated laboratory tests to exclude glycosuria. 

### 3.5. Sensor Activity at Different pH Values

**Figure 6:**
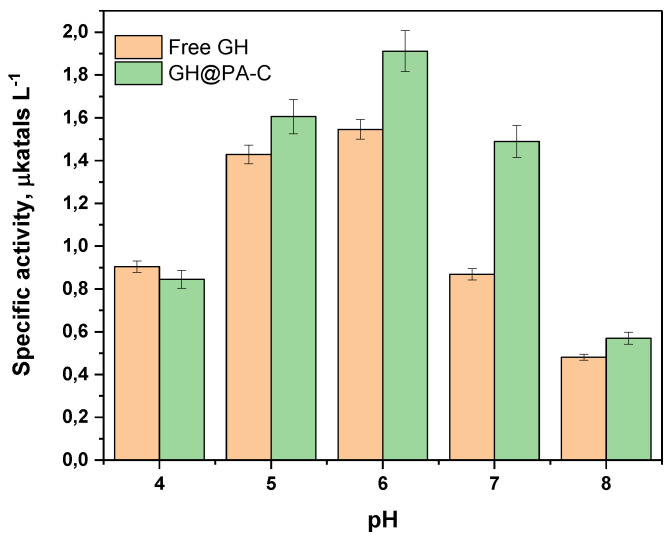
GH and GH@PA-C specific activities as a function of pH in the 4–8 interval. Results obtained at 23 °C. Normalization by the total protein content was performed to enable comparison.

The pH value of human urine can vary in a relatively broad range, between 4.6 and 8.0, due to factors such as diet, hydration levels and certain medical conditions [31]. It is therefore essential to investigate the effect of pH on the activity of the new biosensor. Figure 6 presents a comparison of the specific activities of the free GH dyad and the GH@PA-C complex in the 4–8 pH range. As seen in this figure, the maximum specific activity values were obtained in slightly acidic conditions at pH = 5–6, with the MP-supported dyad performing statistically better than the free enzymes. The pH values of 4 and 8 are out of the optimal range for both GOx and HRP enzymes, but they are still operational, and the free enzymes and the GH@PA-C complex have similar activities. 

### 3.6. Detection of Glucose in Urine

**Figure 7:**
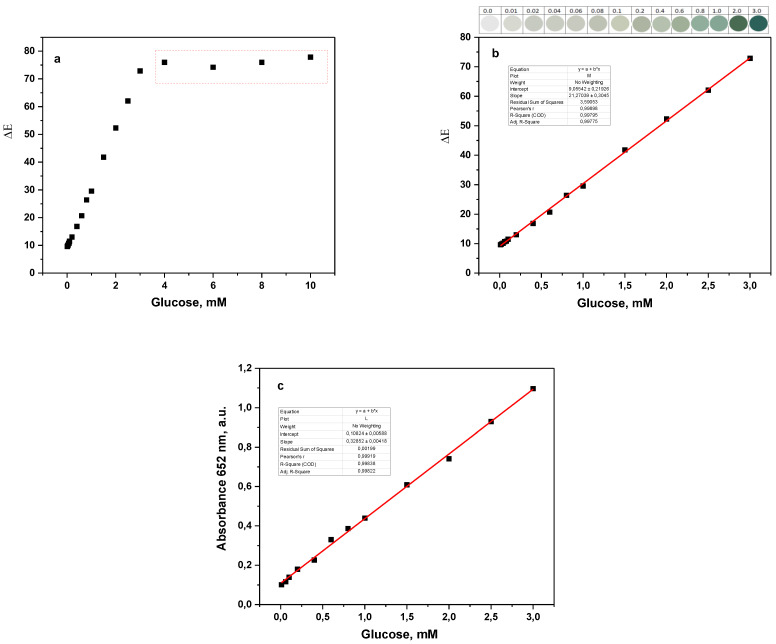
Relationship between the GH@PA-C sensor´s color change and the glucose concentration in human urine determined by (**a**,**b**) processing of smartphone digital photographs; (**c**) UV/VIS measurement. The colors of the sensor areas in (**b**) are after the detection of the respective glucose concentrations. More statistical data from the linear regression are given in Table S1 of the Supplementary Materials.

Bearing in mind the pH dependency of the GOx/HRP cascade activity, GH@PA-C-based biosensors were used to detect and quantify glucose in human urine samples by applying both smartphone image analysis (Figure 7a,b) and UV/VIS spectral data (Figure 7c). For this purpose, urine with pH 5.6 was collected from a healthy person, and glucose was added to sample to cover the concentration range of 0.01−10 mM. All analyses were performed at room temperature. 

In contrast to the experiments with aqueous glucose solutions, in the case of urine testing for both the image treatment and UV/VIS measurements, the linear response was in the entire interval from 0.01 to 3.00 mM of glucose, with the coefficients of linear regression being above 0.995. The LOD values determined were 30.7 µM and 53.7 µM for the CIELab treatment and UV/VIS, respectively. The LOD value was calculated from the relation 3s/k, where *s* is the standard deviation of the blank sample, and *k* denotes the slope of the calibration curve. More numerical and statistical data for the standard calibration curve in urine are given in Table S1 of the Supplementary Information. Figure 7b also shows the color of the interrogation area after the detection of different glucose concentrations from digital photographs. Some basic characteristics of biosensors using the same GOx/HRP/TMB system reported in the literature are listed in Table 2. 

From Table 2, it can be seen that the parameters of the GH@PA-C biosensor in this study are comparable to and generally better than those reported earlier for similar optical biosensors. The time for the stable color change completion in our case is only 20 s compared to 5–10 min for the systems reported previously, which could be an advantage in its practical use. The new sensor displays a wide linear interval, both for healthy concentrations (as low as 0.01 mM, i.e., 0.2 mg/dL) and in the higher range of 1.5–3.0 mM (i.e., 27–54 mg/dL), typical for unhealthy glycosuria. Another essential feature of the new GH@PA-C sensor that distinguishes it from many of the existing ones is that it represents an all-inclusive system, bearing all three necessary elements, namely, the GOx and HRP enzymes, as well as the TMB chromogenic substrate, which is ultimately essential for a quick and accurate glucose determination in POCT.

More comparative data for glucose sensor parameters are presented in Table S2 of the Supplementary Materials.

### 3.7. Shelf Life and Multiple Uses of the Biosensor

Storage stability was investigated by keeping the sensors in a dark and cool place for one month and then measuring their performance in comparison to freshly prepared ones. Urine samples from a healthy person (pH 5.6) with undetectable glucose (i.e., below 0.01 mM) were used. For the purpose of the test, glucose was added to it to give three different concentrations of glucose: 0.04 and 0.4 mM, typical for a healthy person, and 2.0 mM (person with glycosuria). Based on the image treatment and the calculated ΔE, the glucose concentration read by the sensor was determined from the standard calibration plot in Figure 7b. From Table 3, it can be seen that the recovery and RSD values of fresh sensors (samples 1, 2 and 3) and those stored for 30 days at −8 °C (samples 4, 5 and 6) were quite good, with the relative dispersion being slightly higher for the lowest concentration range of the stored samples. Even after 30 days of storage, the sensors still reacted extremely rapidly when in contact with the urine samples, producing stable colors within 20 s. 

During this experiment, an interesting observation was made. About 15–20 min after the test, the blue-green color of the sensors started to fade, and after 30 min, these sensors were impossible to distinguish from the non-used ones with the naked eye. This observation prompted us to validate whether or not these colorless and already-used sensors could be reused again several consecutive times. The results in Table 3 for four additional urine tests with samples 4–6 30 min after fading showed that this was possible. The recovery and RSD percentages after the fifth application grew slightly, with this effect being more pronounced for the sensors used in the sample with 0.04 mM glucose. Nevertheless, the three sensors continued to be operational after the fifth consecutive application. To the best of our knowledge, this possibility for multiple uses of GOx/HRP/TMB-based glucose sensors for urine has never been reported as yet. The fact that the color of the sensor disappears means that the blue charge-transfer complex [TMB…DTMB] (see Figure S3) decomposes, producing free TMB and TMB^2+^. Apparently, the latter is reduced back to TMB, thus recovering the GOx/HRP/TMB system for additional use. This is possible only because the three components of the sensor are in close vicinity to one another on the PA MP support. Of course, further research is needed to prove this explanation, but the important finding in this case is that the new GH@PA-C biosensors for glucose can be used repeatedly, which could open the way for novel POCT devices and procedures. 

## 4. Conclusions

This is the first report on a very fast acting, multiple-use optical biosensor for glucose detection in urine samples. It is based on the GOx/HRP enzyme dyad supported on PA6 microparticles and also integrates the TMB chromogen. The specific localization of GOx and HRP on the porous microparticulate support allowed for (i) high activities of the immobilized enzymes, comparable to that of the free dyad, and (ii) the very fast action of the biosensor, with the stabilization of the color change within only 20 s. In human urine samples, the new sensor showed an area of linearity between 0.01 and 3.0 mM glucose (or 0.2–54.8 mg/dL), as determined by the CIELab treatment of digital photographs, with a LOD of 30.7 µM. The biosensor showed high operational activity in the pH range of 4–8 and good detection performance after 30 days of storage. A unique feature of the new sensor is the possibility of its multiple uses within a 30 min time period without the serious deterioration of its parameters. The results of this study show that the new glucose biosensor is a promising candidate for the on-site, fast, non-invasive, user-friendly and accurate detection of glucose in urine samples. 

## Figures and Tables

**Table 1:** Activity parameters of the GH@PA-R and GH@PA-C bienzyme complexes compared to the free GOx/HRP enzymes.

Sample	Total Protein, mg	Slope, Abs s^−1^ (×10^4^)	Specific Activity ^a^, mol L^−1^ s^−1^ (×10^7^)	Specific Activity, µkatals.L^−1^	Specific Activity, U.L^−1^	Relative Activity, %
GH Free	0.01750	389 ± 5	9.97692	0.99769	59.861	100.0
GH@PA-R	0.00501	183 ± 6	4.68974	0.46897	28.138	47.0
GH@PA-C	0.00499	372 ± 5	9.52564	0.95256	57.154	95.5

^a^ TMB substrate extinction coefficient ε = 39,000 M^−1^cm^−1^; slope determined in the 0–15 s interval.

**Table 2:** Comparison of glucose biosensor parameters from previously reported works.

Quantification	LOD	Linear Range	Response Time	Analyte	Ref.
UV/VIS	0.2 µM	0.5–50 µM	-	Urine	[15]
Digital image	15.6 µM	0.5–300 µM	15 min	Urine	[16]
UV/VIS Digital image	0.45 mM	1–11 mM	5 min	Urine, water	[17]
Digital image UV/VIS	49 µM 28 µM	25–200 µM	-	Water	[18]
Digital image	0.14 mM	0.25–10 µM	-	Water	[19]
UV/VIS Thermal	0.012 mM 0.049 mM	0.05–10 mM	-	Water	[20]
Digital image UV/VIS	30.7 µM 64.9 µM	0.01–3.0 mM	20 s	Urine	This study

**Table 3:** Detection of glucose in real human urine samples by CIELab digital image processing. Samples 1–3: freshly prepared sensors; samples 4–6: sensors used 30 days after their production, first application. Samples 4–6 were then applied in 4 additional consecutive tests (rounds-2–4) separated by 30 min periods. The recovery and RSD data are based on 3 measurements for each concentration.

Sample	No. of Uses	Glucose Added mM	Detection Mean ± SDD mM *	RSD ** n = 3 %	95% Confidence Interval, mM ***	Recovery, n = 3 %
1		2.00	1.9033 ± 0.0308	1.616	1.9033 ± 0.0348	95.163
2	1st	0.40	0.4076 ± 0.0076	1.863	0.4076 ± 0.0086	101.898
3		0.04	0.0458 ± 0.0006	1.417	0.0458 ± 0.0007	114.584
4		2.00	2.0812 ± 0.0194	0.930	2.0812 ± 0.0219	104.060
5	1st	0.40	0.4002 ± 0.0117	2.920	0.4002 ± 0.0133	100.042
6		0.04	0.0402 ± 0.0041	10.200	0.0402 ± 0.0046	100.576
4		2.00	2.0438 ± 0.0221	1.083	2.0438 ± 0.0251	102.193
5	2nd	0.40	0.4089 ± 0.0262	6.427	0.4089 ± 0.0297	102.219
6		0.04	0.0402 ± 0.0008	21.000	0.0402 ± 0.0100	100.579
4		2.00	2.0928 ± 0.0579	2.766	2.0928 ± 0.0655	104.642
5	3rd	0.40	0.4138 ± 0.0251	6.068	0.4138 ± 0.0284	103.451
6		0.04	0.04276 ± 0.012	29.114	0.0428 ± 0.0141	106.898
4		2.00	2.1028 ± 0.0461	2.1923	2.1028 ± 0.0522	105.141
5	4th	0.40	0.4290 ± 0.0237	5.525	0.4290 ± 0.0268	107.256
6		0.04	0.0442 ± 0.099	22.418	0.0442 ± 0.0112	110.553
4		2.00	2.1428 ± 0.0807	3.766	2.1428 ± 0.0913	107.138
5	5th	0.40	0.4174 ± 0.0138	3.299	0.4174 ± 0.0156	104.351
6		0.04	0.0427 ± 0.0022	5.222	0.0427 ± 0.0025	106.811

* SDD = standard deviation distance (n = 3); ** RSD = relative standard deviation; *** confidence level 95%, α = 0.05; n = 3.

## Data Availability

The data in this research are presented in the manuscript and in the Supplementary Materials.

## Supplementary Materials

The following supporting information can be downloaded at https://www.mdpi.com/article/10.3390/polym15132802/s1. Figure S1: Chemical reactions occurring during AAROP of ε-caprolactam to PA6 MPs; Figure S2: Chemical structure of Pebax^®^ MH1657; Figure S3: Schematic presentation of the GOx/HRP/TMB cascade reaction for glucose determination; Figure S4: Overall catalytic activities of free GH dyad, GH@PA-R and GH@PA-C complexes—complete numerical data; Table S1: Detection of glucose in urine—standard curve statistics; Table S2: Comparison of glucose biosensor parameters in previously reported works, according to Ref. [17].

## References

[1] Vyas T., Choudhary S., Kumar N., Joshi A., Gogoi M., Patra S., Kundu D. (2022). Point-of-Care Biosensors for Glucose Sensing. Nanobiosensors for Point-of-Care Medical Diagnostics.

[2] McMillan J.M., Walker H.K., Hall W.D., Hurst J.W. (1990). Blood Glucose. Clinical Methods: The History, Physical and Laboratory Examinations.

[3] Karpova E.V., Shcherbacheva E.V., Galushin A.A., Vokhmyanina D.V., Karyakina E.E., Karyakin A.A. (2019). Noninvasive Diabetes Monitoring through Continuous Analysis of Sweat Using Flow-Through Glucose Biosensor. Anal. Chem..

[4] Lee H., Song C., Hong Y.S., Kim M., Cho H.R., Kang T., Shin K., Choi S.H., Hyeon T., Kim D.H. (2017). Wearable/disposable sweat-based glucose monitoring device with multistage transdermal drug delivery module. Sci. Adv..

[5] Gao Z.F., Sann E.E., Lou X., Liu R., Dai J., Zuo X., Xia F., Jiang L. (2018). Naked-eye point-of-care testing platform based on a pH-responsive superwetting surface: Toward the non-invasive detection of glucose. NPG Asia Mater..

[6] Jang S., Xu C. (2018). Review of emerging approaches in non- or minimally invasive glucose monitoring and their application to physiological human body fluids. Int. J. Biosen. Bioelectron..

[7] Bruen D., Delaney C., Florea L., Diamond D. (2017). Glucose Sensing for Diabetes Monitoring: Recent Developments. Sensors.

[8] Haxha S., Jhoja J. (2016). Optical based non-invasive glucose monitoring sensor prototype. IEEE Photon. J..

[9] Nery E.W., Kundys M., Jeleń P.S., Jönsson-Niedziółka M. (2016). Electrochemical Glucose Sensing: Is There Still Room for Improvement?. Anal. Chem..

[10] Corrie S.R., Coffey J.W., Islam J., Markey K.A., Kendall M.A.F. (2015). Blood, sweat, and tears: Developing clinically relevant protein biosensors for integrated body fluid analysis. Analyst.

[11] Zhu W.J., Feng D.-Q., Chen M., Chen Z.D., Zhu R., Fang H.L., Wang W. (2014). Bienzyme colorimetric detection of glucose with self-calibration based on tree-shaped paper strip. Sens. Actuators B Chem..

[12] Xu K., Chen X., Zheng R., Zheng Y. (2020). Immobilization of multienzymes on support materials for efficient biocatalysis. Front. Bioeng. Biotechnol..

[13] Ge J., Lei J., Zare R.N. (2012). Protein–inorganic hybrid nanoflowers. Nat. Nanotechnol..

[14] Braz J.F., Dencheva N.V., Malfois M., Denchev Z.Z. (2023). Synthesis of Novel Polymer-Assisted Organic-Inorganic Hybrid Nanoflowers and Their Application in Cascade Biocatalysis. Molecules.

[15] Sun J., Ge J., Liu W., Lan M., Zhang H., Wang P., Wang Y., Niu Z. (2014). Multi-enzyme co-embedded organic–inorganic hybrid nanoflowers: Synthesis and application as a colorimetric sensor. Nanoscale.

[16] Ariza-Avidad M., Salinas-Castillo A., Capitán-Vallvey L.F. (2016). A 3D mPAD based on a multi-enzyme organic–inorganic hybrid nanoflower reactor. Biosens. Bioelectron..

[17] Luo X., Xia J., Jiang X., Yang M., Liu S. (2019). Cellulose-Based Strips Designed Based on a Sensitive Enzyme Colorimetric Assay for the Low Concentration of Glucose Detection. Anal. Chem..

[18] Hosu O., Lettieri M., Papara N., Ravalli A., Sandulescu R., Cristea C., Marrazza G. (2019). Colorimetric multienzymatic smart sensors for hydrogen peroxide, glucose and catechol screening analysis. Talanta.

[19] Luo M., Li M., Lu Y., Xia M., Zhao Q., Wang D. (2022). In-situ growth of multienzyme-inorganic hybrid nanoflowers on PVA-co-PE nanofibrous strip for colorimetric biosensor. Colloids Surf. A Physicochem. Eng. Asp..

[20] Sun X., Li Y., Yang Q., Xiao Y., Zeng Y., Gong J., Wang Z., Tan X., Li H. (2021). Self-assembled all-inclusive organic-inorganic nanoparticles enable cascade reaction for the detection of glucose. Chin. Chem. Lett..

[21] Tang Z., Li X., Tong L., Yang H., Wu J., Zhang X., Song T., Huang S., Zhu F., Chen G. (2021). A Biocatalytic Cascade in an Ultrastable Mesoporous Hydrogen-Bonded Organic Framework for Point-of-Care Biosensing. Angew. Chem. Int. Ed..

[22] Hosu O., Ravalli A., Piccolo G.M.L., Cristea C., Sandulescu R., Marrazza G. (2017). Smartphone-based immuno-sensor for CA125 detection. Talanta.

[23] Dencheva N., Denchev Z., Lanceros-Méndez S., Ezquerra T.E. (2016). One-step in-situ synthesis of polyamide microcapsules with inorganic payload and their transformation into responsive thermoplastic composite materials. Macromol. Mater. Eng..

[24] Dencheva N., Braz J., Nunes T.G., Oliveira F.D., Denchev Z. (2018). One-pot low temperature synthesis and characterization of hybrid poly(2-pyrrolidone) microparticles suitable for protein immobilization. Polymer.

[25] Cano-Raya C., Dencheva N.V., Braz J.F., Malfois M., Denchev Z.Z. (2020). Optical biosensor for catechol determination based on laccase-immobilized anionic polyamide 6 microparticles. J. Appl. Polym. Sci..

[26] Ashiotis G., Deschildre A., Nawaz Z., Wright J.P., Karkoulis D., Picca F.E., Kieffer J. (2015). The fast azimuthal integration Python library: pyFAI. J. Appl. Crystallogr..

[27] Bartczak Z., Galeski A., Argon A.S., Cohen R.E. (1996). On the plastic deformation of the amorphous component in semicrystalline polymers. Polymer.

[28] Josephy P.D., Eling T., Mason R.P. (1982). The horseradish peroxidase-catalyzed oxidation of 3,5,3′,5′-tetramethylbenzidine. Free radical and charge-transfer complex intermediates. J. Biol. Chem..

[29] Misono Y., Ohkata Y., Morikawa T., Itoh K. (1997). Resonance raman and absorption spectroscopic studies on the electrochemical oxidation processes of 3,3′,5,5′-tetramethylbenzidine. J. Electroanal. Chem..

[30] Lin J.L., Palomec L., Wheeldon I. (2014). Design and analysis of enhanced catalysis in scaffolded multienzyme cascade reactions. ACS Catal..

[31] Luo A.M., Shao Y., Zhang K.J., Wang Y.W., Peng Y. (2017). Syntheses of three terbium complexes as fluorescent probes and their application on the pH detection of routine urine test. Chin. Chem. Lett..

