# Supplementary material for: Fast, Multiple-Use Optical Biosensor for Point-of-Care Glucose Detection with Mobile Devices Based on Bienzyme Cascade Supported on Polyamide 6 Microparticles
Source: Polymers (Basel). 2023 Jun 24;15(13):2802. doi: 10.3390/polym15132802 (PMC10346291; doi:10.3390/polym15132802)
Supplement: Supplementary file 1 [file polymers-15-02802-s001.zip › polymers-2452662-supplementary.pdf]

Fast, Multiple-Use Optical Biosensor for Point-of-Care Glucose Detection with Mobile Devices Based on Bienzyme Cascade Supported on Polyamide 6 Microparticles

Joana F. Braz [1], Nadya V. Dencheva [1,*], Shafagh D. Tohidi [2] and Zlatan Z. Denchev [1,*]

[1] IPC-Institute for Polymers and Composites, University of Minho, Guimarães 4800-056, Portugal; joanabraz@dep.uminho.pt
[2] DTx CoLab-Digital Transformation CoLab, University of Minho, Guimarães 4800-056, Portugal; shafagh.tohidi@dtx-colab.pt
* Correspondence: nadiad@dep.uminho.pt; denchev@dep.uminho.pt

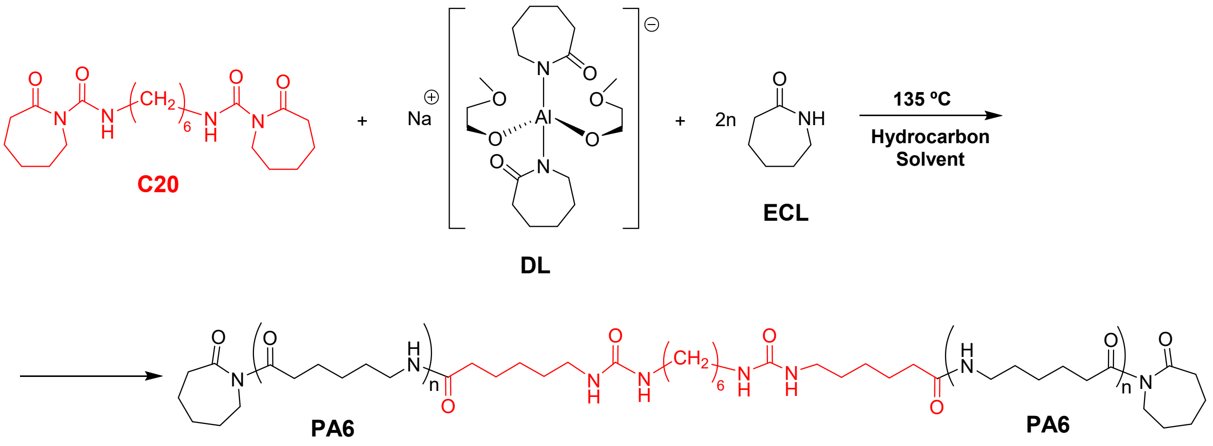

Figure S1. Chemical reactions occurring during AAROP of laurolactam (R1 = $(CH_2)_5$) to neat PA6 MP. The active substance of the AAROP activator is designated as C20; The chemical structure of the AAROP initiator dicaprolactamato-bis-(2-methoxyethoxo)-aluminate (DL) is presented wherein R = $OCH_2CH_2OCH_3$.

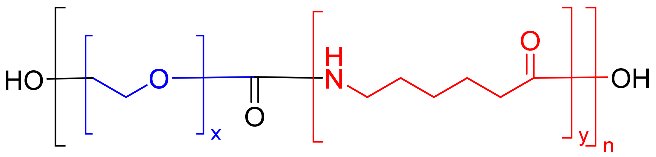

Figure S2. Chemical structure of Pebax® MH1657: a segmented polyether-block-polyamide 6 copolymer wherein x/y =60/40 wt.%

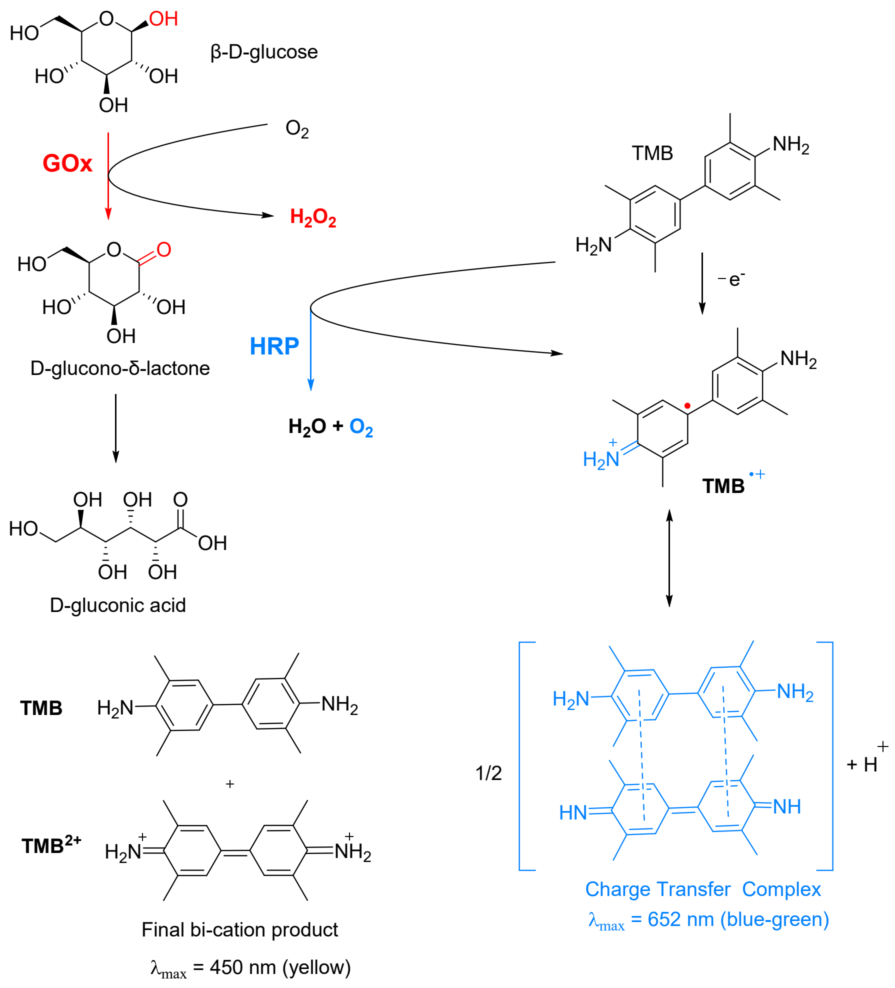

Figure S3. Schematic representation of the bienzyme cascade reaction used for β-D-glucose colorimetric determination. The TMB structures are presented according to [1,2]. GOx = glucose oxidase; HRP = horseradish peroxidase; TMB = 3,3′,5,5′-tetramethlbenzidine; $TMB^{\bullet+}$= cation-free radical derivatve of TMB; DTMB = diimine derivative of TMB; TMB^{2+} = bication of TMB.

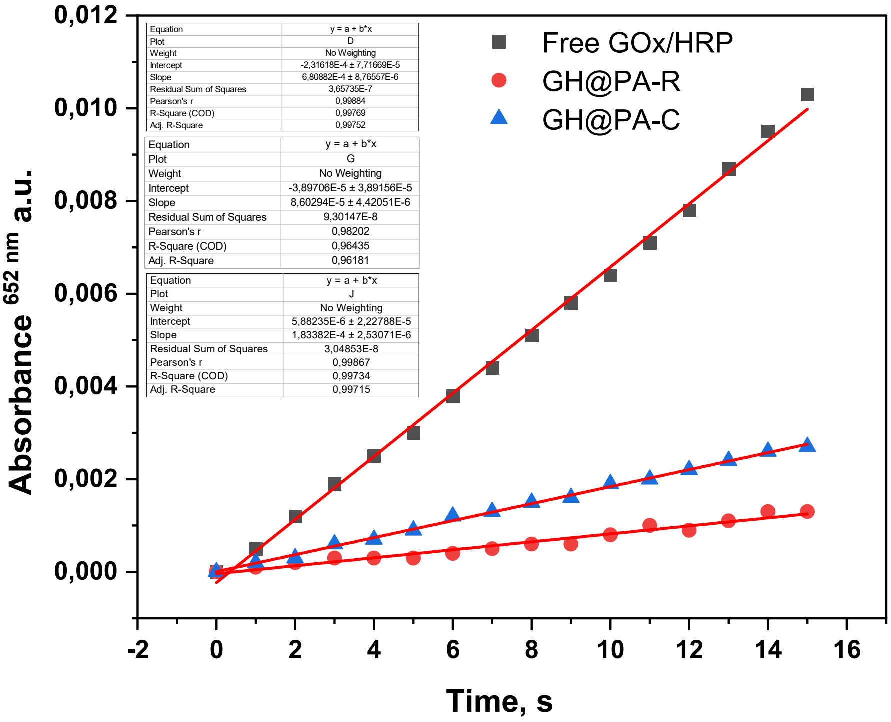

Figure S4. Overall catalytic activity of free GH dyad, GH@PA-R and GH@PA-C complexes – complete numerical data

Table S1. Detection of glucose in urine – standard curve statistics (**Figure 7b)**

N	Glucose concentration C_A, mM	ΔE mean value	SDD n=3
1	0.01	9.641	0.2289
2	0.02	9.843	0,2129
3	0.04	10.152	0.2046
4	0.06	10.671	0.2204
5	0.08	10.874	0.3126
6	0.1	11.518	0.3246
7	0.2	13.040	0.3591
8	0.4	16.853	0.4906
9	0.6	20.712	0.5021
10	0.8	26.374	0.6792
11	1	29.571	0,8287
12	1.5	41.820	1,2255
13	2	52.321	1,5369
14	2.5	62.060	1,8462
15	3	72.866	2,0186

Addditional statistics data:

SDD = standard deviation distance (n = 3)

Linear regression: $\Delta E = m^*C_A + b$

Slope, m = 21.27039 ± 0.3045

Intercept, b = 9.05542 ± 0.21926

Residual Sum of Squares = 3.59053

Regression of regression $R^2 = 0.99795$

Degree of freedom = 13

Standard Deviation about the regression, $S_r = 0.5255$

Confidence interval, CI (Confidence level = 95%)

Standard deviation of the Slope, S_m= 0.6532 (95% CI, $\alpha = 0.05$; t = 2.145)

Standard deviation of Intercept, $S_b = 0.4703$ (95% CI, $\alpha = 0.05$; t = 2.145)

Table S2. Comparison between glucose biosensor parameters from previously reported works according to Ref. [3]

Analyte	Method	LOD	Linear range	Response time	Media	Ref.
glucose	colorimetric	0.025 mg/mL	0.1-3000 μg/mL	30 min	-	[4]
	colorimetric	0.8 μM	5-500 μM	40 min	-	[5]
	colorimetric	84 AU/mM	0.1-1.0 mM	15 min	-	[6]
	colorimetric	0.28 μM	0-400 μM	30 min	-	[7]
	colorimetric	3 μM	5-70 μM	65 min	-	[8]
	colorimetric	-	-	30 min	-	[9]
	colorimetric	0.3 mM	1-11 mM	-	-	[10]
	colorimetric	-	0-5 mM	30 min	-	[11]
	colorimetric	0.5 mM	0.5-100 mM	10 min	-	[12]
	colorimetric	0.45 mM	1-11 mM	5 min	-	[3]

References

1. Josephy, P.D.; Eling, T.; Mason, R.P. *J. Biol. Chem.* **1982**, *257*, 3669-3675.
2. Misono, Y.; Ohkata, Y.; Morikawa, T.; Itoh, K. *J. Electroanal. Chem.* **1997**, *436*, 203-212.
3. Luo, X.; Xia, J.; Jiang, X.; Yang, M.; Liu, S. Anal. Chem. 2019, 91:15461−15468.
4. Shen, W.; Sun, J.; Seah, J. Y. H.; Shi, L.; Tang, S.; Lee, H. K. Anal. Chim. Acta 2018, 1001, 32−39.
5. Wang, Q.; Zhang, X.; Huang, L.; Zhang, Z.; Dong, S. ACS Appl. Mater. Interfaces 2017, 9, 7465−7471.
6. Gabriel, E.; Garcia, P.; Lopes, F.; Coltro, W. Micromachines. 2017, 8, 104.
7. Jin, L.; Meng, Z.; Zhang, Y.; Cai, S.; Zhang, Z.; Li, C.; Shang, L.; Shen, Y. ACS Appl. Mater. Interfaces 2017, 9, 10027−10033.
8. Gao, Y.; Wu, Y.; Di, J. Spectrochim. Acta, Part A 2017, 173, 207−212.
9. Riccardi, C. M.; Mistri, D.; Hart, O.; Anuganti, M.; Lin, Y.; Kasi, R. M.; Kumar, C. V. Chem. Commun. 2016, 52, 2593−2596.
10. Zhu, W.-J.; Feng, D.-Q.; Chen, M.; Chen, Z.-D.; Zhu, R.; Fang, H.-L.; Wang, W. Sens. Actuators, B 2014, 190, 414−418.
11. Chaplan, C. A.; Mitchell, H. T.; Martinez, A. W. Anal. Methods 2014, 6, 1296−1300.
12. Ornatska, M.; Sharpe, E.; Andreescu, D.; Andreescu, S. Anal. Chem. 2011, 83, 4273−4280.
